# Awareness, service use and barriers to sexual and reproductive health among adolescents in the Arsi Zone, Oromia, Ethiopia

**DOI:** 10.4102/phcfm.v18i1.5500

**Published:** 2026-08-31

**Authors:** Derese T. Demissie, Rose M. Mmusi-Phetoe

**Affiliations:** 1 Department of Health Studies, College of Human Sciences, University of South Africa, Pretoria, South Africa

**Keywords:** adolescent, awareness, sexual, reproductive health, utilisation

## Abstract

**Background:**

Sexual and reproductive health (SRH) services are crucial for adolescents, yet utilisation remains low in the Arsi Zone of Ethiopia. Identifying the factors affecting adolescents’ use of SRH services is important to address the problem.

**Aim:**

To explore and describe adolescents’ awareness and barriers to SRH services in Arsi Zone, Ethiopia, and improve SRH service use, ultimately reducing preventable SRH-related illness and death among adolescents.

**Setting:**

The study was conducted at six districts of Arsi Zone, Oromia, Ethiopia.

**Methods:**

A school-based cross-sectional study was conducted among 651 randomly selected adolescents aged 15–19 years in Arsi Zone of Ethiopia, from 01 January 2024 to 29 February 2024. Data were collected using a self-administered structured questionnaire. A multivariable logistic regression model was employed to analyse the data. Measures of association were expressed as adjusted odds ratios (AORs) with corresponding 95% confidence intervals (CIs).

**Results:**

This study revealed that being involved in SRH discussions with trusted individuals was significantly associated with SRH utilisation, with better odds (AOR = 2.12; 95% CI: 1.22–3.67). Absence of fear when seeking care was significantly associated with higher utilisation of SRH services (AOR = 2.07; 95% CI: 1.12–3.83). The presence of an SRH problem, such as a sexually transmitted infection (STI), was strongly associated with SRH service utilisation (AOR = 4.88; 95% CI: 2.26–10.54). In addition, the perceived convenience of service operating hours was significantly associated with higher SRH service utilisation (AOR = 3.19; 95% CI: 1.57–6.52).

**Conclusion:**

The findings highlight a significant gap between awareness and use of SRH services among adolescents. This emphasises the need for targeted interventions to improve awareness and access to SRH services for adolescents.

**Contribution:**

The study provides empirical evidence on Ethiopian adolescents’ SRH, addressing gaps in service access, structural and sociocultural barriers, and service use. Its findings offer actionable insights for designing, implementing and evaluating adolescent SRH policies and programmes and contribute to the academic literature.

## Introduction

An adolescent is an individual aged 10–19 years, marking a transition from childhood to adulthood. Adolescence is a unique stage of human development marked by rapid physical, emotional and social changes.^1^ Globally, there are 1.2 billion adolescents, with nearly 90% living in low- and middle-income countries.^2^ Ensuring sexual and reproductive health (SRH) during this period is critical, as adolescents are vulnerable to risky behaviours, sexually transmitted infections (STIs), early marriage and unintended pregnancy.^3,4^ The WHO defines reproductive health as complete physical, mental and social well-being in all aspects related to the reproductive system.^5^ Adolescent SRH services include family planning, STI prevention and treatment, cervical cancer screening, infertility care and maternal health services. However, access is often constrained by cultural norms, lack of confidentiality, negative provider attitudes and limited community support.^6,7^ In Ethiopia, adolescents constitute 26.1% of the population.^8^ Yet, SRH service utilisation remains low, with youth-friendly service (YFS) coverage ranging from 9% to 55%.^9,10^ Barriers include poor awareness, stigma, lack of parental communication and inadequate provider competency.^11,12^ These challenges contribute to high rates of teenage pregnancy, unsafe abortion and STIs among Ethiopian adolescents.^13,14^ Improving adolescent SRH service utilisation is essential to protect against sexual violence, human immunodeficiency virus (HIV), early marriage and unsafe abortion. Accordingly, this study sought to quantitatively identify and measure the determinants of adolescent SRH service utilisation in Southeast Ethiopia, with the overarching goal of generating evidence to guide the development and enhancement of youth-friendly health services.

## Research methods and design

### Study design

A school-based cross-sectional study design was employed to assess adolescents’ SRH services awareness, utilisation and barriers in six randomly selected secondary schools in the Arsi Zone of Ethiopia from 01 January 2024 to 29 February 2024.

### Study setting

The study was conducted in six secondary schools in the Arsi Zone of Oromia Regional State, Ethiopia, located about 175 km south-east of Addis Ababa, with Asella as the zonal administrative centre. In 2022, the Arsi Zone had an estimated population of 3 872 030, of whom 50.4% were male and 49.6% female; adolescents and youth made up about 35%. During data collection, 97 622 students were enrolled in secondary education (Grades 9–12), including 44 734 (45.8%) females and 52 888 (54.2%) males. The six study schools were Lemmu, Hachalu, Bilalo, Iteya, Huruta and Bulala secondary schools.

### Study population and sampling strategy

The study population comprised adolescents aged 15–19 years in six selected secondary schools in the Arsi Zone, Oromia, Ethiopia. A multi-stage random sampling approach was used to obtain the study participants. Schools were selected using simple random sampling from the list of all public secondary schools in six selected districts. A sampling frame was obtained from the Arsi Zone education office, and schools were selected by lottery method. A list of all eligible adolescents in each school was used as a sampling frame. The participants were then proportionally divided by school into 9th, 10th, 11th and 12th grade groups based on the sample size. The total sample size was then proportionally divided among the secondary schools based on the number of adolescents enrolled at each school. The systematic random sampling (SRS) method was applied to pick the study respondents from the attendance list. If the chosen student was absent from the survey, the researcher proceeded to the next student on the list of registered students.

### Inclusion criteria

Adolescents who were attending public secondary school in the six selected districts of Arsi Zone, Oromia, Ethiopia. Adolescents (girls and boys) aged 15–19 years attending secondary school were included in this study.

### Exclusion criteria

Adolescents with serious illnesses or cognitive impairments were excluded from participating. Adolescents or guardians who refuse to consent or assent to participate, and adolescents who are either physically or mentally unwell and unable to engage in reading, writing or listening.

### Sample size determination

The researcher used the single-population proportion formula to calculate the sample size for the study (Equation 1):


$$n=\frac{{\left(Z_{a/2}\right)}^{2}*P(1-P)}{d^{2}}$$ [Eqn 1]

As,

*n* = sample size of the study

*Z* = standard normal variate at the 95% confidence level (i.e. 1.96)

*d* = margin of error (0.05)

*p* = A previous study suggests that the anticipated rate of adolescents’ utilisation of SRH services is 26.1%.^15^

A design effect of two was applied because the study used multi-stage sampling to select respondents, and an additional 10% was included for non-response due to the sensitive nature of the questionnaires, resulting in the final sample size:


$$n=\frac{{\left(1.96\right)}^{2}*0.26(1-0.261)}{{\left(0.05\right)}^{2}}$$

*n* = 296*2 (design effect) = 592

*n* = with 10% non-response rate = 592 + 59 = 651. Therefore, the final sample size for the study was 651.

### Operational definition

#### Awareness

In this study, awareness is defined as respondents having heard about the existence of SRH in their region. A respondent was considered aware if he or she answered ‘Yes’ to the question ‘Have you heard about sexual and reproductive health services (SRH)?’ and was able to mention at least one component of SRH services. Those who answered ‘No’ or could not mention a component were classified as unaware.

#### Utilisation

The operational definition of the use of SRH services was that the use of at least one SRH service was self-reported 6 months before the survey. Measurement was made using a two-step method: Firstly, respondents were asked if they had used an SRH service over the past 6 months, and those who responded positively were further asked to specify the services they had used. Secondly, only respondents who answered ‘Yes’ and mentioned at least one specific SRH service were classified as ‘users’, while those who answered ‘No’ or did not mention any service that was used were classified as ‘non-users’. The 6-month recall period was selected to minimise recall bias while capturing the recent behaviour of adolescents with service use.

#### Barriers

In this study, barriers to the use of SRH services were operationally defined as any perceived or real challenges reported by adolescents that prevented them from accessing or using SRH services. The respondent was classified as having encountered a barrier if he answered ‘Yes’ to the question ‘Have you ever faced a challenge in getting SRH service’? and could mention at least one specific challenge he encountered.

#### Unsafe sexual practice

In this study, unsafe sexual practice was defined by their involvement in sexual behaviour that causes adolescents to be infected with STIs, HIV and acquired immunodeficiency syndrome (AIDS) or unintended pregnancy. This variable was evaluated only by sexually active respondents, those who have confirmed having had sex. Sexual activity was first established using the question ‘Have you ever had sex? Subsequently, unsafe sexual behaviour was determined based on two behavioural indicators: the use of condoms and the age at which sexual start. Respondents were classified as having unsafe sex if they never used condoms during sex, or if they had sex for the first time before the age of 18, or both. Respondents who regularly used condoms and had their first sexual experience at the age of 18 years or over were classified as having safe sex.

### Data collection

Data collection was done using an open-ended, structured, self-administered questionnaire provided to adolescent participants. The school officials were also asked to confirm the information provided by the University of South Africa (UNISA) Health Studies Research Ethics Committee by signing it. This information sheet provides comprehensive details on the study aims and the ethical principles followed. Once permission was granted, an SRS technique was applied in the first phase, following clustering by grade. To facilitate comprehension among participants, the English questionnaire was translated into Afan Oromo, which is the local language. After that, it was reverse-translated to English to check for uniformity. A sample of 651 secondary school adolescents was selected for the study and completed a self-administered questionnaire after providing consent. Parental or guardian permission was obtained for those under 18 years. Assistance was given to adolescents who needed help. Trained healthcare workers collected the data after the questionnaire was pre-tested on 5% of the sample, that is, 33 adolescents. The questionnaire, developed in line with the objective of the study and WHO’s recommendations for adolescent health, was reviewed by the family health experts. The questionnaire’s reliability was also assessed using pilot study data. To establish the reliability of the research instrument, a reliability analysis was conducted, and Cronbach’s alpha coefficient was calculated to be ≥ 0.7 to confirm internal consistency.

### Data analysis

Questionnaires were first reviewed for completeness, then systematically coded, cleaned, and edited before being entered into Statistical Package for the Social Sciences (SPSS) version 30 (IBM Corp., Armonk, NY, United States [US]) for statistical analysis. Descriptive statistics were used to summarise the data, including frequency distributions, means and standard deviations. Binary logistic regression was employed to examine associations between explanatory and outcome variables and to estimate crude odds ratios (COR) with corresponding 95% confidence intervals (CI). Variables with a *p*-value < 0.25 in the bivariate analyses were included in a multivariable logistic regression model to evaluate the independent effect of each predictor on SRH service utilisation, while controlling for potential confounders using backward stepwise elimination. Statistical significance was defined as *p* < 0.05. All assumptions for logistic regression were assessed before finalising the model. Multicollinearity was evaluated using the Variance Inflation Factor (VIF), and no predictor exceeded the common threshold of 10. Global model fit, assessed with the Hosmer–Lemeshow test (*p* > 0.05), indicated adequate agreement between observed and predicted probabilities.

### Ethical considerations

Ethical clearance to conduct this study was obtained from the University of South Africa College of Human Science Ethics Committee (No. 17171555_CREC_CHS2023) and Oromia Regional Health Bureau (No. BFO/DHB/TFA/176/61,07/11/2015 E.C) and Arsi Zonal Education Office (No. W/F/G/A/D72/7986). Additionally, formal permission was obtained from the Arsi Zonal Education Office to facilitate data collection from the educational institution in question. The participants completing the self-administered questionnaire were informed about the study’s objectives. Consent for participating in the study was obtained from all sampled adolescents and parents or guardians, where necessary.

## Results

### Socio-demographic characteristics of the study participants

The study sample comprised 651 adolescents aged 10–19 years enrolled in secondary schools. The overall response rate was 100%. The gender distribution was approximately balanced. Most participants were unmarried (96%). Parental educational attainment was generally low, with most parents having completed only primary education and a minority having attained secondary education (Table 1).

**TABLE 1:** Demographic characteristics of the adolescents in Arsi Zone, Oromia, Ethiopia, 2024 (*N* = 651).

Variables	*n*	%
**Age (years)**		
15–16	305	46.9
17–19	346	53.1
**Gender**		
Female	331	50.8
Male	320	49.2
**Educational level**		
Grade 9	144	22.1
Grade 10	272	41.8
Grade 11	149	22.9
Grade 12	86	13.2
**Marital status**		
Single	625	96.0
Married	21	3.2
Divorced	5	0.8
**Adolescent living arrangement**		
Live independently	36	5.5
Both parents	360	55.3
Father only	54	8.3
Mother only	179	27.5
Guardian or relative	22	3.4
**Father’s educational level**		
No formal education	162	24.9
Primary school Grade (1–8)	286	43.9
Secondary school Grade (9–12)	161	24.7
College (diploma and above)	42	6.5
**Mother’s educational level**		
No formal education	149	22.9
Primary school Grade (1–8)	323	49.6
Secondary school Grade (9–12)	146	22.4
College (diploma and above)	33	5.1

### Adolescents’ awareness and utilisation of sexual and reproductive health services

This study found that 80.8% (*n* = 526) of adolescents were aware of at least one SRH service, with family planning being the most recognised. However, actual utilisation remained markedly low at 23.5% (*n* = 153), indicating a substantial awareness-utilisation gap. This disparity suggests that awareness alone is insufficient for service uptake, and that structural, sociocultural and individual barriers continue to impede adolescents’ effective access to SRH services.

### Information sources and preferred channels for awareness of sexual and reproductive health services among adolescents in Arsi Zone, Oromia, Ethiopia, 2024

Healthcare workers were the predominant source of SRH information (70%), whereas social media was the least frequently reported source (35.9%). Consistently, healthcare workers were also reported as the most preferred source of SRH information (74.9%), while television and radio were the least preferred (20.7%). Notwithstanding this relatively high level of awareness, actual utilisation of SRH services was considerably lower, at 23.5% (*n* = 153), thereby demonstrating a substantial gap between awareness and service use. This divergence suggests that awareness alone is insufficient to guarantee service uptake and implies the presence of persistent structural, sociocultural and individual-level barriers that limit adolescents’ effective access to and utilisation of SRH services (Table 2).

**TABLE 2:** Sources and preferred source of sexual and reproductive health services awareness among adolescents in Arsi Zone, Oromia, Ethiopia, 2024 (*N* = 651).

Sources of SRH information for adolescents	Sources from which adolescents received SRH information		Preferred sources for adolescents to receive SRH information	
	*n*	%	*n*	%
Healthcare worker	368	70.0	394	74.9
School teacher	322	61.2	311	59.1
Social media	189	35.9	118	22.4
Television or radio	219	41.6	109	20.7
Parents	315	59.9	174	33.1
Peer educators	133	25.3	176	33.5

SRH, sexual and reproductive health.

### Sexual and reproductive health services accessed by adolescents

**FIGURE 1:**
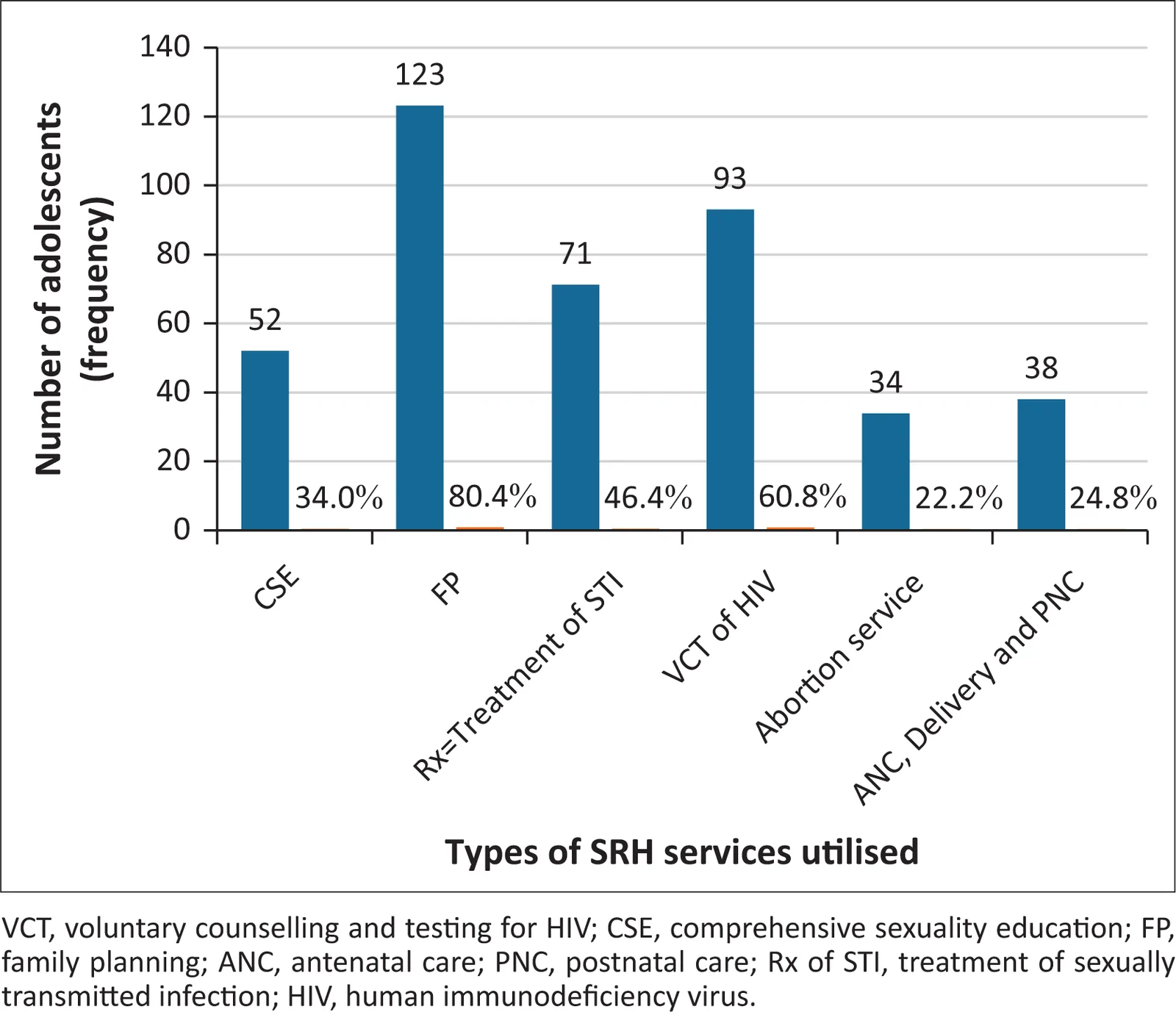
Sexual and reproductive health services utilised by adolescents in Arsi Zone, Oromia, Ethiopia, 2024 (*N* = 651).

Among adolescents who accessed SRH services, family planning (80.4%) and HIV voluntary counselling and testing (60.8%) constituted the most frequently utilised service categories, whereas antenatal or postnatal care (24.8%) and abortion services (22.2%) exhibited the lowest levels of uptake (Figure 1). With respect to service delivery points, private health facilities represented the predominant source of care (47.7%, *n* = 73), followed by government facilities (37.9%, *n* = 58), while youth-friendly centres showed the lowest utilisation (14.4%, *n* = 22). Taken together, these utilisation patterns indicate that adolescent engagement with SRH services is primarily oriented towards preventive and diagnostic interventions obtained through private sector facilities, whereas maternal health services, abortion-related care and youth-dedicated service platforms remain substantially underutilised. This distribution may reflect the combined effects of social stigma, restrictive legal and policy environments, constrained availability of appropriate facilities and adolescents’ preference for the perceived confidentiality afforded by private providers over the accessibility of purpose-designed YFSs.

### Adolescent autonomy to decide on sexual and reproductive health service utilisation

**FIGURE 2:**
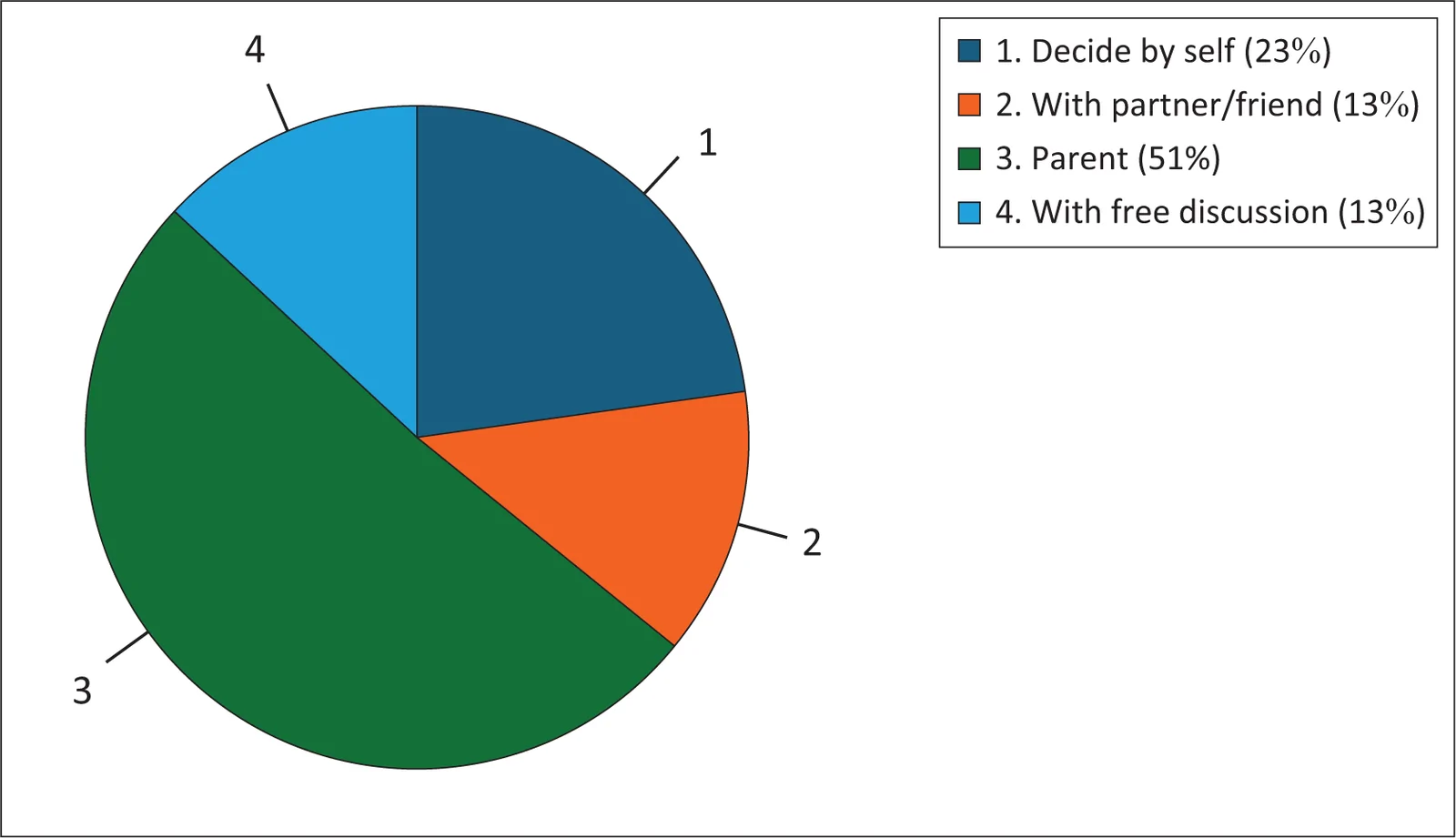
Adolescent autonomy in decision-making for sexual and reproductive health services in Arsi Zone, Oromia, Ethiopia, 2024 (*N* = 651).

Parental influence was identified as the predominant determinant in adolescent SRH decision-making (51.0%, *n* = 332), whereas autonomous decision-making was least frequently reported (23.0%, *n* = 150) (Figure 2). This marked discrepancy indicates a substantial limitation in adolescents’ autonomy regarding their own SRH choices, potentially reinforcing reliance on parental approval and, in turn, discouraging adolescents, particularly those residing in more restrictive household environments, from independently accessing services they perceive as being socially or culturally disapproved of by their families.

### Onset of sexual activity, awareness of sexual health risks, and determinants of high-risk sexual behaviours among adolescents in Arsi Zone, Oromia, Ethiopia

Among the 651 adolescents surveyed, 27.8% (*n* = 181) reported a history of sexual intercourse, with a mean age at sexual debut of 16.4 years (range: 11–19). This distribution indicates that a substantial proportion initiated sexual activity below the legal age of consent in Ethiopia. Among sexually active adolescents, awareness of the adverse outcomes associated with unsafe sexual practices was high. The most frequently identified potential consequences were unplanned pregnancy (78.6%, *n* = 449), school withdrawal (78.5%, *n* = 448), HIV and other STIs (72.9%, *n* = 416) and cervical cancer (50.8%, *n* = 290). Despite the high level of risk awareness, peer pressure was identified as the predominant factor contributing to engagement in unsafe sexual practices (63.9%, *n* = 417), followed by alcohol and other substance use (13.4%, *n* = 87), exposure to pornography and social media (8.0%, *n* = 52), cultural norms (7.5%, *n* = 49), sexual coercion (5.1%, *n* = 33) and transactional sex (2.0%, *n* = 13). The continued prevalence of unsafe sexual practices in the context of substantial risk awareness underscores a pronounced knowledge-behaviour paradox, in which social and environmental determinants appear to supersede cognitive risk recognition as proximal drivers of adolescent sexual risk-taking.

### Adolescents’ barriers to use sexual and reproductive health services

**FIGURE 3:**
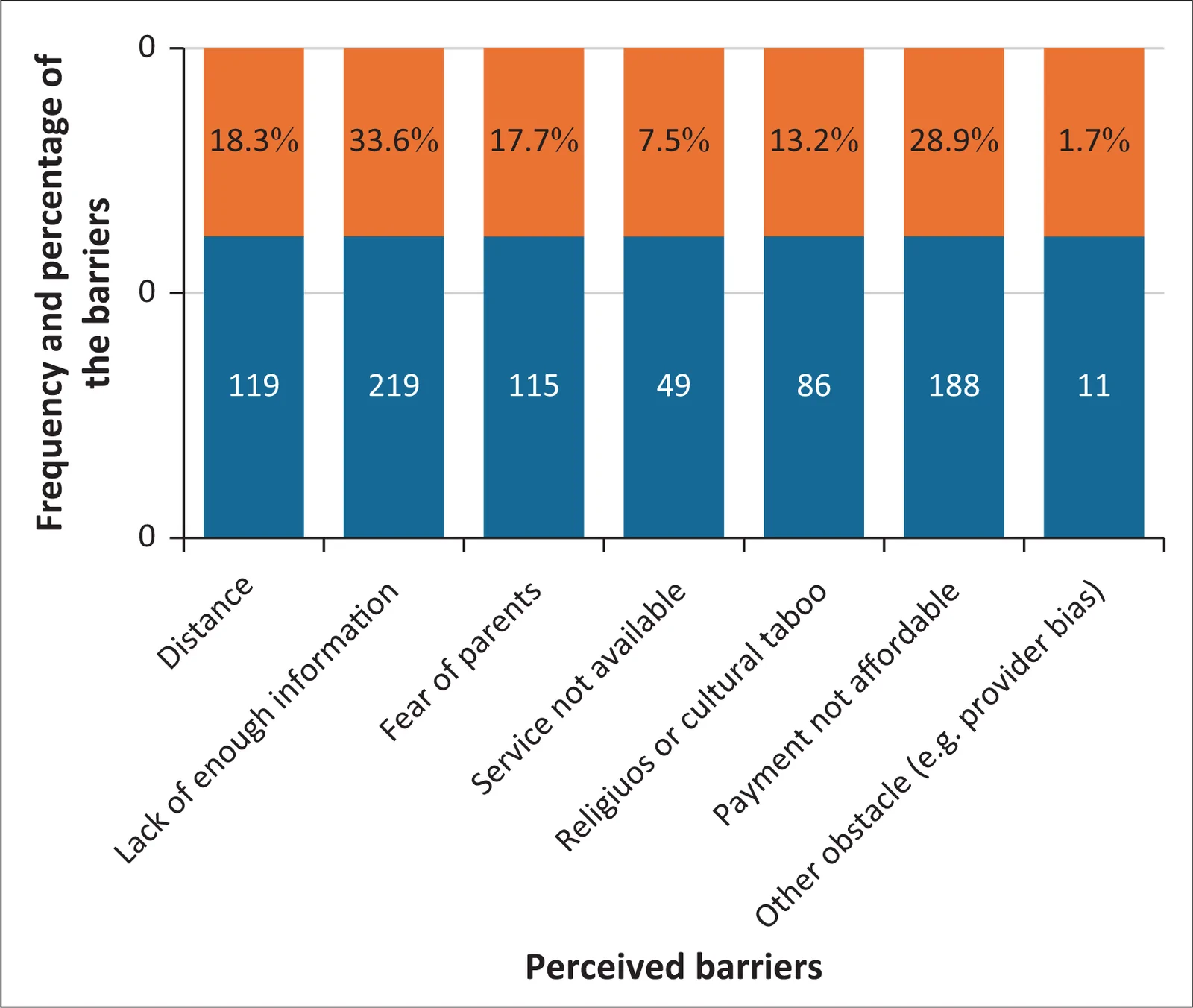
Barriers perceived by adolescents that hinder the utilisation of sexual and reproductive health services in Arsi Zone, Oromia, Ethiopia, 2024 (*N* = 651).

A substantial proportion of adolescents (40.4%, *n* = 263) reported experiencing multiple barriers to the utilisation of SRH services. Among these, insufficient information regarding the availability of services emerged as the most frequently cited barrier (33.6%, *n* = 219), whereas provider bias was the least commonly reported (*n* = 11) (Figure 3). The predominance of information-related barriers, relative to logistical constraints such as cost (18.3%) and distance (17.7%), indicates that deficiencies in health communication and outreach constitute the principal impediments to adolescents’ access to SRH services in the study setting.

### Factors associated with adolescent utilisation of sexual and reproductive health services

Table 3 shows a multivariate regression of adolescents who discussed SRH topics with parents, teachers, or healthcare workers in the past six months, who are more likely to use SRH services (AOR = 2.12, 95% CI: 1.22–3.67). Absence of fear from family and other community when accessing SRH services (AOR = 2.07, 95% CI: 1.12–3.83), adolescents with SRH problems (AOR = 4.88, 95% CI: 2.26–10.54), and those with access to convenient YFS centre operating hours were (AOR = 3.19, 95% CI: 1.57–6.52) more likely to use SRH services (Table 3).

**TABLE 3:** Factors associated with adolescent utilisation of sexual and reproductive health services in Arsi, Oromia, Ethiopia, 2024 (*N* = 651).

Variables	Categories	SRH utilisation		COR	95% CI	Reference group	AOR	95% CI	*p*-value
		Yes	No						
Gender	Female	99	232	2.10	1.44–3.06	1	1.10	0.61–1.99	0.745
	Male	54	266						
Educational level	9–10	81	335	0.55	0.38–0.79	1	0.75	0.45–1.24	0.256
	11–12	72	163						
Adolescents get pocket money	Yes	67	139	2.01	1.38–2.93	1	0.74	0.43–1.28	0.285
	No	86	359						
Discussed SRH in the past 6 months	Yes	66	95	3.22	2.18–4.76	1	2.12	1.22–3.67	0.007**
	No	87	403						
Adolescents have no fear of receiving SRHs	Yes	105	186	3.67	2.49–5.40	1	2.07	1.12–3.83	0.021**
	No	48	312						
Adolescents know the availability of the YFS clinic	Yes	94	184	2.77	1.90–4.02	1	1.27	0.73–2.2	0.400
	No	58	312						
Adolescents face SRH problems like STIs	Yes	40	51	3.10	1.95–4.93	1	4.88	2.26–10.54	0.000**
	No	113	447						
The operating hours of the YFS centre are convenient for adolescents	Yes	90	157	6.19	3.62–10.58	1	3.19	1.57–6.52	0.001**
	No	19	205						

Note: 1 = Reference group.

CI, confidence interval; YFS, youth-friendly service; SRH, sexual and reproductive health; COR, crude odds ratio; AOR, adjusted odds ratio.

** , There is an association.

## Discussion

### Discussed sexual and reproductive health

Adolescents who discussed SRH with parents, teachers or healthcare workers were over twice as likely to use SRH services (AOR = 2.12, 95% CI: 1.22–3.67), underscoring interpersonal communication as a key driver of health-seeking. This effect extends beyond information sharing: dialogue with trusted adults reduces shame and stigma, while building adolescents’ self-efficacy and skills to navigate services. Studies from Southern Ethiopia and Ghana show that adolescents in communicative relationships with parents, providers and teachers are significantly more likely to use SRH services, and that lack of such dialogue predicts non-use.^16,17,18^ A study from south-western Uganda further shows that the quality of communication, its openness, non-judgement, and consistency, determines its impact.^19^ In Ethiopia, where adolescent sexuality is highly stigmatised, and SRH discussion in families and schools is limited, these conditions are rarely met, creating an information vacuum that leaves adolescents unable to identify, trust or access services. Thus, barriers to SRH use are not only structural but also communicative, rooted in norms that silence adolescent sexuality and block interpersonal pathways to care. These findings call for SRH programmes to move beyond service provision and invest in adolescents’ communicative environments: integrating structured, age-appropriate SRH dialogue into school curricula, training teachers and healthcare workers as consistent, comfortable communication partners, and engaging parents through community programmes that frame SRH discussion as a protective responsibility rather than a cultural violation.

### Fear of social exposure

Adolescents who did not fear being seen by family or community members while seeking SRH services were over twice as likely to use them (AOR = 2.07, 95% CI: 1.12–3.83). This shows that social surveillance is a structural barrier to adolescent health-seeking, not just a matter of individual privacy. In many settings, seeking SRH care is socially read as proof of sexual activity, exposing adolescents to reputational damage, family conflict and community stigma that may seem to outweigh the benefits of care. Studies from Ethiopia, Rwanda and Laos similarly show that fear of parental discovery is a persistent barrier to adolescent SRH service use across sub-Saharan African and Asian contexts.^15,20,21^ This convergence suggests a widespread tension between adolescents’ health needs and adult surveillance where adolescent sexuality is morally regulated. While earlier work has focused on parental disapproval, the present findings also emphasise community-level surveillance by neighbours and other community members as an equally important deterrent. In the Arsi Zone, stigma therefore operates both within households and across the wider community, embedded in everyday social interactions. In Ethiopia, where religious and community institutions reinforce social cohesion and regulate adolescent behaviour, this dynamic is especially strong for girls. For female adolescents, SRH service use is more often interpreted as sexual permissiveness and moral deviance, adding a gendered layer to stigma and further discouraging service use. These findings call for SRH delivery models that go beyond physical proximity. Services should include adolescent-specific entry points, strong and enforceable confidentiality, comprehensive training of providers in non-judgemental, adolescent-responsive care, and targeted community sensitisation. Such interventions should explicitly detach SRH service use from moral stigma and promote it as a normal, responsible health behaviour deserving social support rather than sanction.

### History of sexual and reproductive health problems

A prior history of SRH problems emerged as the strongest independent predictor of service utilisation in this study (AOR = 4.88, 95% CI: 2.26–10.54), with an effect size that substantially exceeds comparable estimates reported from Ghana and Bahir Dar, Ethiopia,^18,22^ thereby warranting more detailed analytical scrutiny. This disproportionately elevated association likely reflects a high burden of unmet SRH needs in the Arsi Zone, where adolescents appear to seek services predominantly after the onset of acute or severe health problems. This pattern is theoretically congruent with the Health Belief Model, which posits that perceived severity of an experienced health threat exerts a more powerful influence on health-seeking behaviour than anticipatory risk perception alone. Within the Ethiopian context, characterised by limited preventive health-seeking norms, low levels of health literacy, and insufficiently publicised YFSs, adolescents may lack both the requisite knowledge and the sociocultural legitimacy to engage proactively with SRH services. Consequently, service utilisation is often reserved as a measure of last resort rather than integrated into routine health-maintenance behaviours. This predominantly reactive pattern of service use signals a critical shortcoming in preventive SRH programming, indicating that existing YFSs currently function more as crisis-response platforms than as mechanisms for ongoing adolescent health promotion.

### Health facility operating hours

Perceived convenience of health facility hours was a significant structural determinant of SRH service use (AOR = 3.19, 95% CI: 1.57–6.52). Nearly half of adolescents (47.6%, *n* = 224) found opening times inconvenient, while morning was the preferred visiting period (57.3%). This reveals a key mismatch: ‘youth-friendly’ facilities often operate at times that do not align with adolescents’ actual availability, a pattern reported across sub-Saharan Africa and in Nigeria.^23,24^ In Ethiopia, rigid school schedules and heavy household duties during standard working hours mean that time, not motivation, is the main barrier: demand exists, but service delivery is poorly aligned with adolescents’ daily routines. Accordingly, the focus of intervention shifts from individual behaviour change to structural and system-level reform, including extending health facility hours to early mornings and weekends and establishing school-based satellite SRH clinics to reduce scheduling barriers to adolescents’ SRH access.

### Strength

The study employed a relatively large sample size (*N* = 651), which provided sufficient statistical power for multivariable analyses and increased the reliability of the findings among school-attending adolescents. A multi-stage random sampling procedure, involving random selection of schools and systematic selection of participants, was used to minimise selection bias. Conducting the study in a school-based setting facilitated access to a concentrated and heterogeneous adolescent population, reduced non-response, and improved the overall quality of the data. Furthermore, the use of multivariable logistic regression to adjust for potential confounding variables enabled the identification of independent determinants of SRH service utilisation, thereby strengthening the analytical rigour of the study.

### Limitations

The use of a cross-sectional study design restricts the interpretation of findings to associations. The reliance on self-reported data introduces the potential for recall bias and social desirability bias. Furthermore, the sampling frame was limited to school-based populations, which may not be representative of out-of-school adolescents or the broader community. In addition, the geographic scope of the study was confined to the Arsi Zone, thereby limiting the external validity of the findings and constraining their generalisability to other regions of Ethiopia.

## Conclusion

Adolescent use of SRH services in Ethiopia’s Arsi Zone is low, increasing young people’s risk of high-risk sexual behaviour. Key reasons include a lack of comprehensive SRH information. Structural and health-system barriers, such as long distances, few adolescent-friendly services, inconvenient hours, high costs and religious and cultural taboos, further limit trust and access. Fear of being recognised by community members or health workers can completely deter care-seeking. Some factors promote use: adolescents with previous SRH problems are more likely to seek care, indicating reactive rather than preventive use, and those who discuss SRH with parents, teachers or peers use services more, highlighting the role of interpersonal communication and social support. The study underscores the necessity for urgent, targeted interventions to enhance awareness, accessibility and utilisation of SRH services. It further advocates for the implementation, by political authorities and health-sector leadership, of rigorously evidence-based educational strategies and community engagement initiatives aimed at addressing the identified individual, social and structural barriers.
